# Genotypic Differences in Morphological, Physiological and Agronomic Traits in Wheat (*Triticum aestivum* L.) in Response to Drought

**DOI:** 10.3390/plants13020307

**Published:** 2024-01-20

**Authors:** Qingqing Wang, Yi Wu, Suleiman Fatimoh Ozavize, Cheng-Wei Qiu, Paul Holford, Feibo Wu

**Affiliations:** 1 Provincial Key Laboratory of Crop Germplasm, Department of Agronomy, College of Agriculture and Biotechnology, Zijingang Campus, Zhejiang University, Hangzhou 310058, China; 22216152@zju.edu.cn (Q.W.); wy13985514395@163.com (Y.W.); ozavizefatty@yahoo.com (S.F.O.); qiucw@zju.edu.cn (C.-W.Q.); 2 School of Science, University of Western Sydney, Penrith, NSW 2751, Australia; p.holford@westernsydney.edu.au; 3 Jiangsu Co-Innovation Center for Modern Production Technology of Grain Crops, Yangzhou University, Yangzhou 225009, China

**Keywords:** wheat (*Triticum aestivum* L.), drought, agronomic traits, water use efficiency, photochemical efficiency

## Abstract

Drought is one of the main environmental factors affecting crop growth, and breeding drought-tolerant cultivars is one of the most economic and effective ways of increasing yields and ensuring sustainable agricultural production under drought stress. To facilitate the breeding of drought-tolerant wheat, this study was conducted to evaluate genotypic differences in the drought tolerance of 334 wheat genotypes collected from China and Australia with the aim of screening for drought-tolerant and -sensitive genotypes and to elucidate the corresponding physiological mechanisms. A hydroponic-air experiment (roots exposed to air for 7 h/d and continued for 6 d) showed significant genotypic differences in shoot and root dry weights among the genotypes. The relative shoot and root dry weights, expressed as the percentage of the control, showed a normal distribution, with variation ranges of 20.2–79.7% and 32.8–135.2%, respectively. The coefficients of variation were in the range of 18.2–22.7%, and the diversity index was between 5.71 and 5.73, indicating a rich genetic diversity among the wheat genotypes for drought tolerance. Using phenotypic differences in relative dry weights in responses to drought stress, 20 of each of the most drought-tolerant and drought-sensitive genotypes were selected; these were further evaluated in pot experiments (watering withheld until the soil moisture content reached four percent). The results showed that the trends in drought tolerance were consistent with the hydroponic-air experiment, with genotypes W147 and W235 being the most drought-tolerant and W201 and W282 the most sensitive. Significant genotypic differences in water use efficiency in response to drought were observed in the pot experiment, with the drought-tolerant genotypes being markedly higher and the two sensitive genotypes being no different from the control. A marked increase in bound water content in the drought stress plants was observed in the two drought-tolerant genotypes, while a decrease occurred in the free water. The reductions in photochemical efficiencies of PSII, transpiration rates, net photosynthesis rates, chlorophyll contents and stomatal conduction in the drought-sensitive genotypes W201 and W282 under drought stress were higher than the two tolerant genotypes. This study provides a theoretical guide and germplasm for the further genetic improvement of drought tolerance in wheat.

## 1. Introduction

Drought is one of the major natural disasters faced by mankind, and the rapid development of economies and the expansion of populations will directly lead to continuing increases in the size of arid areas brought under production. According to the Food and Agriculture Organization of the United Nations, the world’s highly arid, arid, semi-arid and semi-humid arid areas cover about 6.1 billion hectares of land, accounting for 41% of the Earth’s land area, and it is expected that by the end of the 21st century, global dry areas will exceed half of the total land area [1]. Some countries are likely to have more severe drought problems in the future, including China [2]. According to the Ministry of Water Resources, as of May 2021, China’s cultivated land affected by drought had an area of 29,881 ha, seriously affecting the development of China’s agriculture.

Drought stress leads to decreases in crop yields and quality. When crops are under drought stress, changes in their morphology, physiology and gene expression occur [3]. During drought stress, intracellular water is lost, which causes cell dehydration and disturbances in the plant’s water metabolism. Since water is essential for plant growth and development, drought stress results in a reduction of mitosis and cell division that limits growth. Under mild drought, the leaves of plants atrophy, curl and thin, resulting in a decrease in leaf area [4]. During severe drought, stem growth is significantly reduced, resulting in weak plants and reduced crop growth and yield [5]. Studies have found that in order to adapt to water deficits, roots undergo significant structural changes. For example, plants may increase the number of root vessels to improve water transport [6] and may also have increased root density to promote water absorption [7]. However, a high root–shoot ratio will reduce the water use efficiency, which is not conducive to high yields.

Photosynthesis is the major source of energy for plants and an important physiological process affecting crop growth. Leaves are the main organs in which photosynthesis occurs, and under drought stress plant leaf growth is restricted and stomata close, which affects chlorophyll contents and the absorption of CO_2_. In addition, intercellular CO_2_ concentrations are reduced, the photosystems are perturbed and carbon assimilation is limited, thereby reducing photosynthetic rates [8].

Wheat is one of the three major cereal crops in the world due to its good storage characteristics and high nutritional value, and as one of the main food crops in China, the yield of wheat is closely related to food security [9]. In China, drought has become one of the main natural disasters facing agricultural production, and the drought caused by reductions in rainfall, water shortages or high temperatures has seriously affected wheat yields [10]. With climate change and frequent natural disasters, improving wheat drought resistance has the potential to increase wheat yields in China and elsewhere and enhance the stability and sustainability of food production, thereby making an important contribution to guaranteeing food security. Drought resistance in wheat is a polygenic trait, and there are complex drought resistance mechanisms. One of the most effective ways to mitigate the effects of drought on quality and low yields of wheat under drought stress is to select drought-tolerant germplasm. Therefore, this study was conducted first to identify drought-tolerant and -sensitive wheat germplasm using a hydroponic-air drought treatment [11] and then to assess the performance of selected tolerant and sensitive genotypes under drought stress in pots to aid breeders in developing drought-tolerant wheat varieties.

## 2. Materials and Methods

### 2.1. Preliminary Screening Using a Hydroponic-Air Treatment Simulating Drought

The experimental materials were 334 wheat (*Triticum aestivum* L.) genotypes sourced from China and Australia (Table S1), and the “W” designations are codes for the various accessions. The hydroponic-air drought experiment was conducted in a network room at the Zijingang Campus, Zhejiang University, Hangzhou, China. Aliquots of 30 wheat seeds/genotype (uniform size, no damage, skin intact) were sown into holes in PVC planting boards with gauze mesh at the bottom of each hole. There were 10 × 9 holes per board (480 mm × 360 mm) and five seeds were planted per hole. Each genotype had three replicates of the two treatments, drought and control. The PVC boards were placed on upturned black hydroponic boxes and covered with three layers of wet filter paper and then with plastic film to maintain moisture and temperature. Seven days after sowing, the boards were placed in black hydroponic boxes (480 mm × 360 mm × 170 mm) containing 30 L of water, ensuring the seeds were in direct contact with the water. Twelve days after sowing, wheat seedlings with poor growth were removed and the water was replaced with basic nutrient solution (BNS) (mg/L): (NH_4_)_2_SO_4_, 48.2; MgSO_4_, 65.9; K_2_SO_4_, 15.9; KNO_3_, 18.5; Ca(NO_3_)_2_, 59.9; KH_2_PO_4_, 24.8; Fe-citrate, 5; MnCl_2_·4H_2_O, 0.9; ZnSO_4_·7H_2_O, 0.11; CuSO_4_·5H_2_O, 0.04; HBO_3_, 2.9; H_2_MoO_4_.

Two treatments were initiated at the two-leaf stage (~10 days after sowing): (1) drought stress, in which plant roots were exposed to air for 7 h (9:30–16:30) daily for a period of six days by raising the boards so that the plant roots were out of the solution [11]; and (2) control, in which the plants were maintained in the nutrient solution throughout the experiment. The experiment was laid out in a completely randomized design with three replicates per wheat genotype per treatment. The pH of the solution was adjusted to 5.9 ± 0.1 with NaOH or HCl as required. The nutrient solution was continuously aerated with pumps and renewed every three days.

After six days of drought stress, when there were obvious differences between genotypes, we evaluated the drought tolerance of each genotype according to the degree of seedling wilting using a total of 5 grades, ranging from 0 to 4 (phenotypic score). All leaves wilting among replicates were rated as 0 points, and leaves without wilting among replicates were rated as 4 points (growth normal) [12]. Then plants were taken from each treatment, their shoots and roots separated and their fresh weights recorded; the dry weights were determined following oven-drying for 48 h at 70 ℃. To further evaluate drought tolerance, we calculated an integrated score using the following formula: integrated score = shoot relative dry weight × 0.33 + root relative dry weight × 0.33 + phenotype score × 0.33 [13]. The higher the score, the higher the tolerance.

### 2.2. Pot Selection Experiments

To further evaluate drought resistance and to verify the results of the hydroponic trial, two separate pot selection experiments (One and Two) were conducted on the genotypes selected from the hydroponic trial. There were three replicates per genotype in each experiment, with drought stress and control as the main plot and genotype as the sub-plot. In Experiment One, 40 wheat genotypes with significant differences in drought tolerance (20 of the most drought-tolerant and 20 of the most drought-sensitive) based on the results of hydroponic selection were used. The experiment was carried out in an artificial climate room. The soil in which the seeds were planted was collected from the experimental farm in the West District of Zijingang Campus, Zhejiang University. The soil (450 g) was evenly mixed with BNS (250 mL) two days before sowing, and nine seeds were sown in each pot (pot size: 68 mm × 68 mm × 79 mm). There were two treatments at the two-leaf stage (~10 days after sowing): (1) drought stress, in which the seedlings were subjected to a drought stress for seven days by withholding irrigation until the soil moisture content reduced to around 4% and stayed at this level for three days; and (2) control, in which the soil in each pot was kept at 30–40% water holding capacity throughout. There were three replicates with six plants per replicate. At the end of the drought stress, shoot fresh and dry weights were also measured. To further evaluate drought tolerance, an integrative score was calculated (based on [13]) as follows: integrated score = phenotypic score × 0.25 + shoot relative fresh weight × 0.25 + shoot relative dry weight × 0.25 + relative water content × 0.25.

In Experiment Two, we used the two most drought-tolerant (W147 and W234) and the two most drought-sensitive (W201 and W282) genotypes identified from the hydroponic trial and Experiment One; YM20, a common cultivated wheat variety, was used as a check. Soils were prepared as described for Experiment One. For Experiment Two, 5 L plastic buckets each filled with 4.5 kg of air-dried soil were used, into which 1 L of BNS was added two days before sowing; ten seeds per bucket were sown, and seven days after emergence, the seedlings were thinned to six seedlings per genotype per bucket. To ensure that the amount of water per bucket was consistent, we added 500 mL water per bucket before treatment so that the soil was moist. There were two treatments at the three-leaf stage: (1) control, in which the soil in each bucket was kept moist (30–40% water holding capacity) throughout; and (2) drought stress, in which the seedlings were subjected to drought stress for 45 days until the soil moisture content fell to approximately 4% and continued at this level for three days; the plants were then rewatered and the normal water supply resumed. Water additions were again stopped for 25 days at the jointing stage until the soil moisture content dropped to 4% and continued at this level for three days. Water was then added to restore and maintain a normal water supply until harvest. A hygrometer (model HH2, Delta-T Devices, Cambridge, UK) was used to measure soil moisture contents. During the drought treatments, the soil moisture was measured once a day in the morning and once in the afternoon. After drought stress at the seedling stage, when the soil moisture content fell to approximately 4%, the second leaf from two plants of each genotype was taken to measure water saturation deficit according to Zhao et al. [13]. The fresh weights, air-dry weights and dry weights were determined and the amounts of free and bound water were calculated according to Zhao et al. [13]. Then, shoot heights, fresh and dry weights were measured; each genotype had three replicates. The height of each remaining wheat plant was measured, and the number of effective tillers (tillers producing spikes) was counted at the post-jointing stage. The content of leaf chlorophyll was determined using a Minolta SPAD-502 chlorophyll analyzer. The fluorescence parameters were determined using a porometer/fluorometer (model LI-600; LiCOR). The photosynthetic rate, intercellular CO_2_ concentration, transpiration rate and stomatal conductance were measured using a LI-6800 photosynthesis system. The ratio of the photosynthetic rate to the transpiration rate was used as the water utilization rate. At maturity, the parts above the last internode were harvested, from which spike lengths, spike numbers per plant, kernel numbers per plant, spikelet numbers and grain yields were determined. The seeds were dried in an oven at 30 ℃ to constant weight, and the thousand-grain weights, grain lengths, grain widths and grain length-to-width ratios were determined by scanning analysis with a Wanshen SC-G Automatic Test Analyzer (Wanshen Company, Shanghai, China).

### 2.3. Statistical Analysis

Excel 2019 was used for the preliminary processing and analysis of experimental data. ANOVA and Tukey’s test were used to evaluate the significance of the difference between the drought treatment and control. Correlation analysis was performed by SPSS 26.0 software. Origin (OriginLab, version 9.1) was used for plotting results.

## 3. Results

### 3.1. Hydroponic-Air Treatment Selection

**Figure 1:**
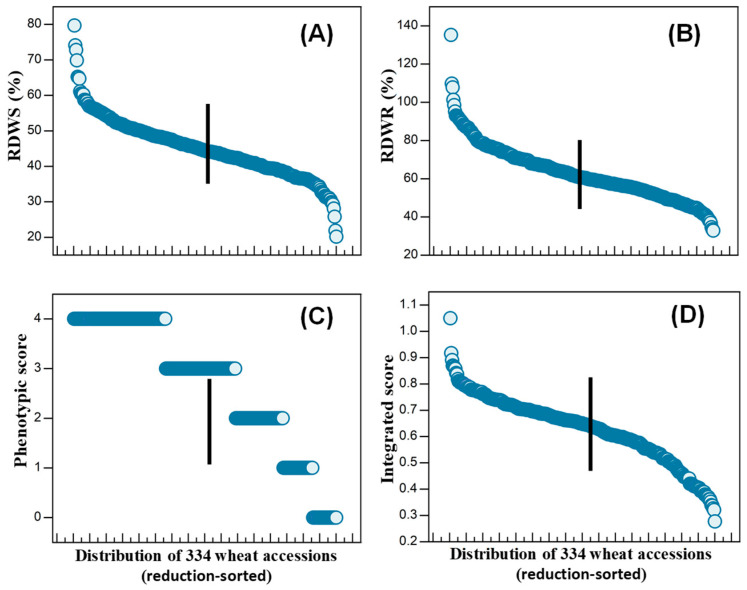
Differences in plant biomass of 334 wheat genotypes due to simulated drought imposed by a hydroponic-air treatment. (**A**,**B**) show the shoot and root dry weights relative to the controls. (**C**) The phenotypic scores based on degree of wilting. (**D**) The integrated scores based on relative shoot and root dry weights and the phenotypic score. In all panels, the data are ranked according to the magnitude of the parameter presented, with the most tolerant on the left and the least on the right. The 20 wheat genotypes with the highest rankings according to the integrated score were considered as drought-tolerant genotypes, and the 20 wheat genotypes with the lowest rankings were considered as sensitive genotypes. The vertical line (“|”) represents the least significant difference at *p* = 0.05 between genotypes.

There were significant differences in shoot and root dry weights among the different treatments and genotypes. The effects of drought stress on the relative shoot and root dry weights and the phenotypic (wilting) and integrated scores of the genotypes are shown in Figure 1 and Table 1. The mean shoot and root dry weights of the 334 genotypes were, respectively, ~45% and 63% lower in the drought treatment than in the control. However, the magnitudes of variation of the data from the control and drought treatments for both the shoots and roots were similar, as judged by the CVs and the Shannon–Weaver diversity indices.

Using the integrated scores, we selected the 20 most drought-tolerant genotypes and the 20 most sensitive genotypes for further selection. The drought-tolerant genotypes chosen were W147, W235, W267, W248, W328, W154, W315, W164, W239, W316, W126, W324, W236, W244, W223, W106, W287, W144, W313, W216—all had scores higher than 0.793 and the genotypes are ranked from most to least tolerant. The sensitive ones chosen were W201, W282, W130, W140, W271, W288, W272, W321, W119, W311, W111, W6, W331, W182, W172, W202, W141, W179, W261, W211; all had scores less than 0.404, and the genotypes are ranked from most to least sensitive (Table S2).

### 3.2. Pot Experiment One

**Figure 2:**
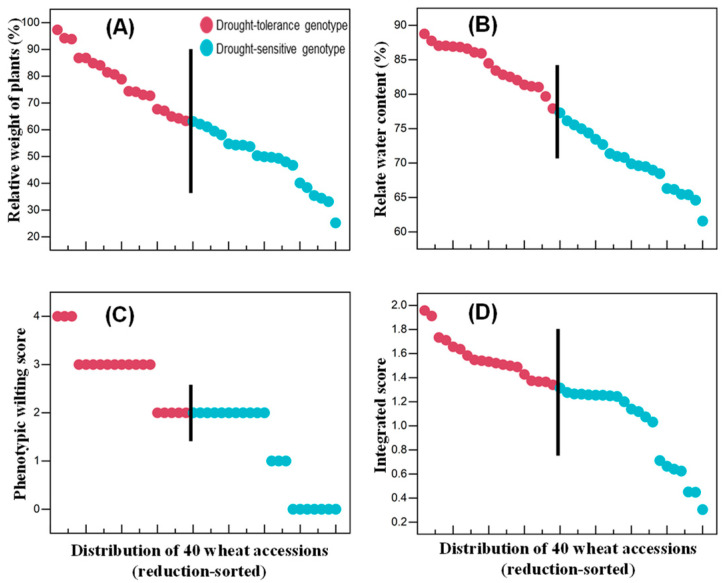
Differences in seedling phenotypes associated with the drought tolerance of the 40 wheat genotypes used in Experiment One. (**A**) Relative shoot weights; (**B**) relative water contents; (**C**) phenotypic scores based on wilting; (**D**) the integrated scores based on relative shoot fresh and dry weights, relative water contents and phenotypic scores. In all panels, the data are ranked according to the magnitude of the parameter presented. The vertical line (“|”) represents the least significant difference at *p* = 0.05 between genotypes. All genotypes reached the two-leaf stage after ten days of growth.

The 40 wheat genotypes selected from the hydroponic trial were subjected to further selection in Experiment One; Table 2 shows the parameters associated with the yields of these plants. The relative shoot fresh and dry weights and relative water contents of the 20 drought-tolerant genotypes were 62.1, 71.2 and 93.8%, respectively, and for the 20 sensitive genotypes they were 50.1, 67.9 and 79.9%, respectively, values that are significantly lower than those of drought-tolerant genotypes (Table 2). In addition, the phenotypic scores showed that the sensitive genotypes exhibited more severe wilting symptoms than the drought-tolerant genotypes (Figure S1). The distributions of the plant weights, relative water contents and the phenotypic and integrated scores of the 40 genotypes are shown in Figure 2A–D. Drought tolerance positively correlated with the integrated score. For the drought-tolerant genotypes, the higher the score, the better the drought tolerance of wheat. Genotypes W147 and W235 showed the least decrease in the above agronomic traits as a result of the drought treatment, and W201 and W282 showed the greatest decrease and were most sensitive to drought stress (Figure 2). Therefore, we selected these genotypes for Experiment Two.

### 3.3. Pot Experiment Two

**Figure 3:**
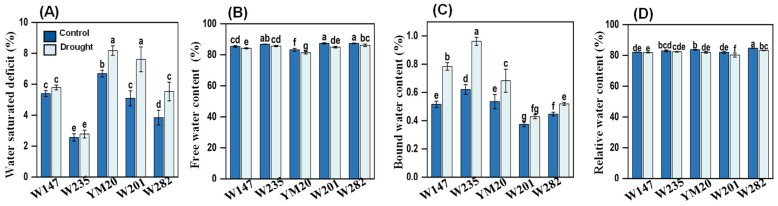
Effects of drought stress on water metabolism of different wheat genotypes. (**A**) Water saturation deficits, (**B**) free water contents, (**C**) bound water contents, (**D**) relative water contents. Data are means ± SEs (n = 3); different letters indicate significant differences at *p* < 0.05 according to Tukey’s tests.

Under drought stress at the seedling stage, the water saturation deficits of W147 and W235 (tolerant genotypes) showed increases of 7.3 and 8.5%, respectively, values that were not significantly different from the controls (Figure 3A). In contrast, the deficits of W201 and W282 (sensitive genotypes) were significantly higher than in the controls, as was the deficit of YM20, the genotype used as a comparator. The deficits of the sensitive genotypes increased the most and were 49.6 and 44.0%, respectively. The increase for YM20 was 22.3%. W235 showed the lowest deficit under both treatment and control conditions of the five genotypes.

The free and relative water contents of the five wheat genotypes were all lower in the droughted plants than in the controls. However, there was little difference in these parameters among the genotypes (Figure 3B,D). In contrast, there were considerable differences in bound water (Figure 3C); the tolerant genotypes had the highest concentrations and the sensitive ones the lowest under both control and drought conditions, and the differences in RWCs between the two treatments were greater for the tolerant types than the sensitive ones. The RWCs of W147 and W235 increased by 52.0 and 55.0% due to drought, whilst the RWCs of W201 and W282 only increased by 14.6 and 16.1%; YM20 was intermediate and increased by 27.7%. As shown in Figure S2A, the water use efficiency of the five wheat genotypes increased to varying degrees under drought stress, but in general, the increases for the drought-tolerant genotypes were significantly higher than for YM20 and the sensitive genotypes. The three parameters related to plant growth, shoot fresh weight (Figure S2B), shoot dry weight (Figure S2C) and plant height (Figure S2D) all showed similar trends. These parameters were all reduced by drought stress, with the reduction being higher for the sensitive types than for the resistant ones. Under both treatment and control conditions, W147 produced the largest biomasses and W282 the lowest; the others were intermediate.

**Figure 4:**
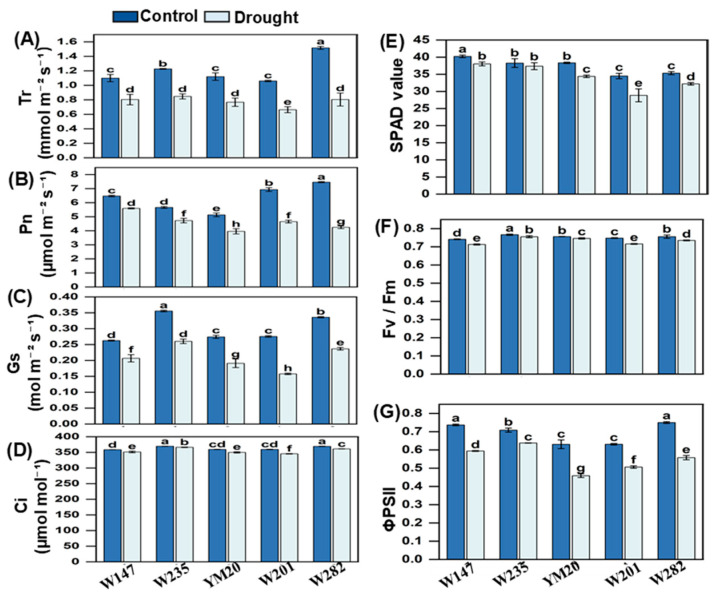
Effects of drought stress on photosynthesis and fluorescence parameters of different wheat genotypes. (**A**) Transpiration rate, Tn; (**B**) net photosynthetic rate, Pn; (**C**) stomatal conductance, Gs; (**D**) intercellular carbon dioxide concentration, Ci; (**E**) SPAD value (chlorophyll content); (**F**) Fv/Fm; (**G**) photochemical efficiency of PSII (ΦPSII). Data are means ± SEs (n = 5); different letters indicate significant differences at *p* < 0.05.

The data related to the physiological parameters are shown in Figure 4A–G; all data sets showed reductions in the parameters measured due to drought stress with, in general, the reductions being greater for the sensitive genotypes than in the tolerant ones; values for YM20 tended to be intermediate. Under drought stress, the values of all seven parameters were lower in the sensitive genotypes than in the two tolerant ones; values for YM20 were either intermediate or similar to the values of the sensitive genotypes. The photochemical efficiency of PSII, transpiration, net photosynthesis, chlorophyll contents and stomatal conduction were the traits most affected by drought and intercellular [CO_2_] and Fv/Fm the least.

**Figure 5:**
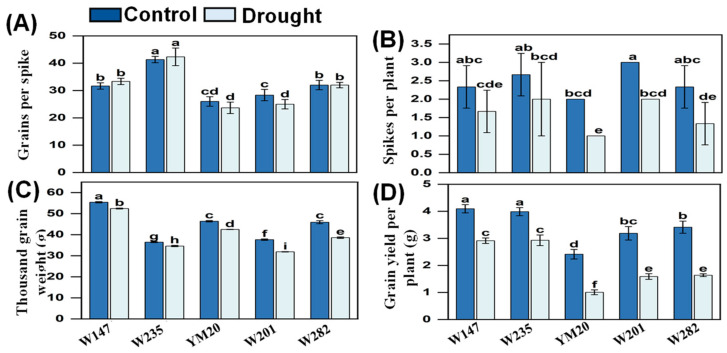
Effects of drought stress on yield parameters of different wheat genotypes. (**A**) Grains per spike; (**B**) spike number per plant; (**C**) thousand-kernel weight; and (**D**) grain yield per plant. Data are means ± SEs (n = 5); different letters indicate significant differences at *p* < 0.05).

**Figure 6:**
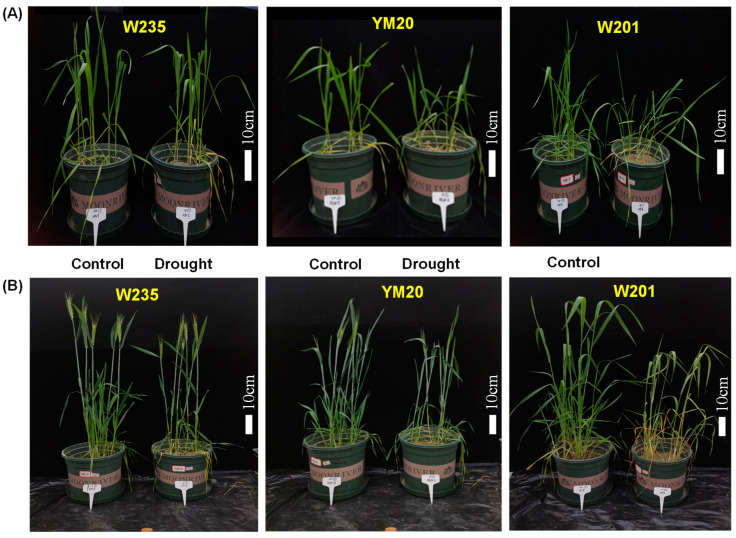
Effects of drought stress on wheat plant phenotypes at seedling (**A**) and maturation (**B**) periods. (**A**) Plant phenotypes at the seedling stage after drought stress had been applied for 45 d by withholding irrigation until the soil moisture content (SMC) reduced to 4%. (**B**) Plant phenotypes during maturation after drought treatments at seedling and jointing stage. At the jointing stage, drought stress was applied for 25 d by withholding irrigation until the SMC reduced to 4%.

The data related to growth parameters are presented in Figure 5A–D and Figure S3 and Table S3, and images of typical plants at the seedling stage and during maturation are presented in Figure 6. With the exception of the length-to-width ratio of the grain, awn length and grains per spike, most other parameters of growth were reduced by drought. The length-to-width ratio of the grain was greater in the two sensitive genotypes under drought conditions but not in the other three genotypes, and there were no differences due to treatment in awn length and grains per spike in the tolerant genotypes and YM20. Again, as for the physiological data, the magnitudes of the growth parameters for the sensitive genotypes were lower than for the tolerant ones. The most affected traits were spikes per plant and yield.

## 4. Discussion

Drought is the main environmental factor limiting wheat production [14], and to mitigate its effects, it is imperative that drought-tolerant crop varieties are produced. Therefore, it is important to be able to easily identify germplasm with good drought tolerance; gaining an understanding of the mechanisms associated with tolerance and susceptibility will facilitate this process. In this study, 334 wheat genotypes from China and Australia were initially screened for tolerance using a hydroponic-air drought stress system—the screen found significant variation in the shoot and root dry weights of the plants that was normally distributed. This is consistent with other studies [15,16] that have shown drought tolerance in wheat to be a polygenic trait controlled by complex tolerance mechanisms. Under drought stress, the relative root dry weights were higher than those of the shoots, showing that drought inhibited shoot growth but promoted root growth, resulting in increases in the root–shoot ratios. Previous research has shown that the length, weight and area of the roots and shoots of plants are inhibited to varying degrees under drought stress [17], and the root system can be directly regulated by the degree of drought stress [18]. In order to survive drought conditions, plants alter their root systems to better obtain water and nutrients from the soil. The roots sense drought stress and stimulate the transport of ABA to leaves so that the whole plant responds [19]. When subjected to drought, plants can change the direction of root growth [20] and the distribution of lateral roots [21], increase root growth and reduce branch growth [22] to adjust their root–shoot ratios and optimize root structure to help withstand the stress conditions. Research has found that ABA signals activate SnRK2 protein kinase, which mediates the phosphorylation of sucrose transporters (SWEET11 and SWEET12) in the phloem to promote the long-distance transport of sucrose from the shoot to the root system, thereby increasing the root–shoot ratio of plants and drought resistance [23].

We selected the 20 most drought-tolerant and sensitive genotypes from the hydroponic-air experiment by calculating an integrated score based on Zhao et al. [13] and verified their drought tolerance in a pot experiment. The results of this experiment were in good agreement with those of the hydroponic-air system, which indicates that the two methods gave similar results. Therefore, the use of the hydroponic-air drought simulation can realistically simulate drought and provides a simple method for quickly screening large numbers of genotypes and has been successfully used with wild barley [11]. The combination of hydroponic and pot experiments makes the screening more accurate and reliable. In addition, in the pot experiment, the phenotypes of the sensitive genotypes were more obvious. Hsiao [24] classified the degree of water stress of mesophytes as follows: (1) mild stress, when the water potential is slightly reduced by a few tenths of an MPa or the relative water content is reduced by about 8–10% compared to that of plants provided with a good water supply under moderate evaporation conditions; (2) moderate stress, in which the water potential is decreased further but generally does not exceed 1.2–1.5 MPa, or the relative water content decreases by more than 10% and less than 20%; and (3) severe stress, in which the water potential decreases by more than 1.5 MPa or the relative water content decreases by more than 20%. In our study, compared with the controls, the relative water contents of the sensitive genotypes under drought stress decreased by 20.1%, indicating that they were under severe drought stress. In contrast, the relative water contents of drought-tolerant genotypes only decreased by 6.2%, indicating that they were only mildly stressed and more drought-tolerant than the sensitive genotypes.

The drought tolerance of these 40 wheat genotypes was evaluated using an integrative scoring approach based on shoot weights, relative water contents and symptoms of wilting. Using these integrated scores, we selected W147 and W235, being the most drought-tolerant genotypes, and W201 and W282, being the most sensitive genotypes, to gain a better understanding of the differences among them. The effects of drought stress on their physiology and yield components were examined in a second pot experiment that lasted for the complete duration of their growth period.

Drought stress disrupts the water balance in plants and interferes with normal physiological and metabolic processes [25]. Water contents, relative water contents, water loss rates and transpiration rates are important characteristics affecting the water relations of plants, and the relative water content of wheat under drought stress is positively correlated with grain yields [26,27]. Water in plant cells exists in free and bound forms, and the ratio of these forms is closely related to plant growth and drought resistance. When the ratio of free water to bound water is high, the protoplasm of cells is in a sol state, the metabolism of plants is vigorous and the growth is fast, but resistance to stress is weak. In contrast, when the ratio of free to bound water is low, the cell protoplasm is in a gel state, metabolic activity is low and growth is slow, but resistance to stress is strong [28,29,30,31]. In our results, leaf water saturation deficits and bound water contents increased due to drought, while the relative water contents and free water contents decreased; similar results have been found in Tibetan wild barley [29] and durum wheat [30]. Rascio et al. [31] showed that drought-tolerant cultivars had greater increases in bound water than susceptible ones and commented that bound water contents may be a promising trait with which to evaluate drought sensitivity. The ratios of free water to bound water of the drought-tolerant genotypes, W147 and W235, decreased by 35.1% and 36.4%, respectively, compared with the controls, while those of the sensitive genotypes decreased by 15.2% and 15.1%, respectively. Changes in the ratio of bound to free water may affect the stability of lipids and proteins [32] and contribute to drought tolerance.

Drought stress can inhibit photosynthesis, and studies have shown that photosynthesis in wheat is seriously affected by drought [14]. Drought reduces photosynthetic rates due to both stomatal and non-stomatal factors [33]. Stomatal restrictions of photosynthesis occur due to decreases in stomatal conductance and the closure of stomata, resulting in limiting concentrations CO_2_ and a decrease in photosynthetic rate [34,35]. Non-stomatal restrictions are due to the destruction of chloroplast structures, the degradation of photosynthetic pigments, decreases in quantum efficiency and the activities photosynthesis-related enzymes and ultimately lead to a decline in the ability of a plant’s cells to fix and assimilate CO_2_ [36,37,38,39]. In our study, transpiration rates, net photosynthesis, stomatal conductance, intercellular carbon dioxide concentrations, water use efficiency and chlorophyll contents were all affected by drought stress, but the decreases were significantly lower in the drought-tolerant genotypes than in the sensitive ones, as has been reported by Zhou et al. [40], Shao et al. [41] and Liu et al. [42]. However, in general, the effects on transpiration were greater than those associated with photosynthesis.

Water use efficiency is an important physiological parameter used for evaluating drought tolerance [43]. Our study showed that after drought stress, the water use efficiency of the five wheat genotypes increased to different degrees in all genotypes, but the greatest increases occurred in the two tolerant ones, as was found in the study by Li et al. [33]. Stomatal conductance is more sensitive to soil water concentrations than photosynthesis [44], as was found in this study, as the decreases in transpiration were greater than those associated with photosynthesis. Although photosynthesis was affected to a lesser degree, the perturbations would still have affected yield, and our study showed that the effects of drought on photosynthesis were more severe in the drought-sensitive genotypes.

Yield and its components are the ultimate measures of crop tolerance to drought, and in this study, reductions in yield due to drought in the susceptible genotypes were approximately twice those of the tolerant ones; all components of yield contributed to these differences. In the tolerant genotypes, awn lengths, spikelets per spike and grains per spike were unaffected by the drought treatments, but in general they were reduced in the susceptible ones. The other components of yield were reduced due to drought in all genotypes, but the reductions were less in the tolerant ones, with the most affected trait being spikes per plant. These multiple effects on each of the yield components again reflect the polygenic nature of drought tolerance.

This study has demonstrated the usefulness of the air-hydroponic system for the initial screening of multiple genotypes for their tolerance to drought. The results of this initial screen were validated in subsequent pot trials, the first involving seedlings and the second taking the plants through to maturity. From these trials, genotypes showing a high level of drought tolerance were selected, and the material can be used in breeding programs for developing drought-tolerant wheat lines. The selection process also identified genotypes exhibiting high sensitivity to drought and together with the tolerant types provides an excellent research resource for further studies on the physiology, biochemistry and molecular biology of the mechanisms that drive tolerance and susceptibility to drought, which in turn can be used to inform breeding programs. Previous studies have shown that the reduction in plant biomass as well as a number of other physiological traits such as chlorophyll content, CO_2_ assimilation rate can be used to evaluate the resistance to adversity [45,46,47]. Significant genotype differences in water use efficiency response to drought were observed in the pot experiment, with the drought-tolerant genotypes W147 and W235 being markedly higher and the two sensitive genotypes being no difference over the control. A marked increase in bound water content from drought stress was observed in the two drought-tolerant genotypes, while a decrease occurred in the free water. The reductions of photochemical efficiency of PSII, transpiration rate, net photosynthesis rate, chlorophyll contents and stomatal conduction in the drought-sensitive genotypes W201 and W282 under drought stress was higher over the two tolerant genotypes. This study provides a theoretical guide and prior germplasm for further genetic improvement of drought tolerance in wheat. However, the next step needs to be assessment in the field under various conditions prior to breeding to confirm tolerance or susceptibility. As drought tolerance is polygenic in nature, there is also a need to identify the genes contributing to the trait and their relative importance.

## Figures and Tables

**Table 1:** Effects of drought stress on shoot and root biomasses of 334 wheat genotypes screened in a hydroponic-air drought simulation.

Item	Shoot DW		Root DW		RDWS (%)	RDWR (%)
	Control	Drought	Control	Drought		
Mean	42.90	18.99	13.44	8.20	44.99	62.77
Minimum	21.10	7.80	4.90	2.80	20.15	32.82
Maximum	75.80	32.10	24.30	12.80	79.67	135.24
Diversity index	5.20	5.68	5.64	5.54	5.71	5.73
CV (%)	22.76	20.16	22.69	20.53	18.24	22.66
Significance	**	**	**	**	**	**

RDWS and RDWR: relative dry weight of shoots and roots expressed as the percentage of control. CV: coefficient of variation. ANOVA was used to evaluate the significance of the difference between drought treatment and control. ** = significance at the 0.01 probability level between genotypes.

**Table 2:** Effect of drought stress on the 20 most drought-tolerant and the 20 most sensitive genotypes selected from the hydroponic-air treatment.

Item	Shoot FW (mg)		RFWS	Shoot DW (mg)		RDWS	Relative Water Content		
	Control	Drought	(%)	Control	Drought	(%)	Control	Drought	%
Drought-sensitive genotypes									
Mean	144.16	69.30	50.07	52.58	34.27	67.86	63.06%	50.23%	79.87
Minimum	87.80	36.70	18.45	35.30	17.80	31.99	54.31%	46.00%	71.57
Maximum	198.80	107.00	72.90	78.60	52.80	97.83	72.15%	59.66%	87.30
CV (%)	33.74	28.36	30.94	23.36	26.91	30.33	8.44	6.71	5.16
Diversity index	0.69	0.67	0.67	0.65	0.65	0.67	0.69	0.65	0.67
Drought-tolerant genotypes									
Mean	206.48	126.68	62.09	60.50	42.90	71.21	69.91%	65.58%	93.78
Minimum	130.10	60.90	31.86	40.40	21.40	34.45	60.91%	55.45%	86.15
Maximum	293.70	193.20	96.22	69.90	66.00	99.84	82.14%	79.70%	98.77
CV (%)	18.09	29.16	28.86	14.70	30.30	27.25	8.65	9.72	3.75
Diversity index	0.67	0.69	0.65	0.56	0.65	0.69	0.61	0.61	0.65
Mean of the 40 genotypes	175.32	98.01	56.08	56.55	38.60	69.53	66.48%	57.91%	86.83
Diversity index	1.31	1.47	1.36	1.45	1.42	1.55	1.44	1.45	1.44
Significance	**	**	**	**	**	**	**	**	**

RFWS and RDWS: relative fresh and dry weights of shoots expressed as the percentage of control. CV: coefficient of variation. The one-way ANOVA and Tukey’s tests were used to evaluate the significance of the difference between drought treatment and control. ** = significance at the 0.01 probability level between genotypes.

## Data Availability

Data are contained within the article and supplementary materials.

## Supplementary Materials

The following supporting information can be downloaded at: https://www.mdpi.com/article/10.3390/plants13020307/s1, Table S1: Details of the 334 accessions of wheat germplasm used in this study. Table S2: The twenty most drought-sensitive genotypes (ranked from most to least sensitive) and the twenty most tolerant genotypes (ranked from most to least tolerant) selected from the hydroponic-air drought simulation. Table S3: Effect of drought stress on growth and physiological traits of wheat plants in the pot experiment expressed as reduced (−)/increased (+) percentages of the well-watered controls (Experiment Two). Figure S1: Seedling phenotypes of drought tolerant and sensitive wheat genotypes in the pot experiment one under drought treatment at SMC 4%. Figure S2: Effects of drought stress on agronomic traits and water metabolism of different wheat genotypes. Figure S3: Effects of drought stress on yield parameters of different wheat genotypes.
