# Supplementary material for: Genotypic Differences in Morphological, Physiological and Agronomic Traits in Wheat (Triticum aestivum L.) in Response to Drought
Source: Plants (Basel). 2024 Jan 20;13(2):307. doi: 10.3390/plants13020307 (PMC10820655; doi:10.3390/plants13020307)
Supplement: Supplementary file 1 [file plants-13-00307-s001.zip › plants-2785137-supplementary.pdf]

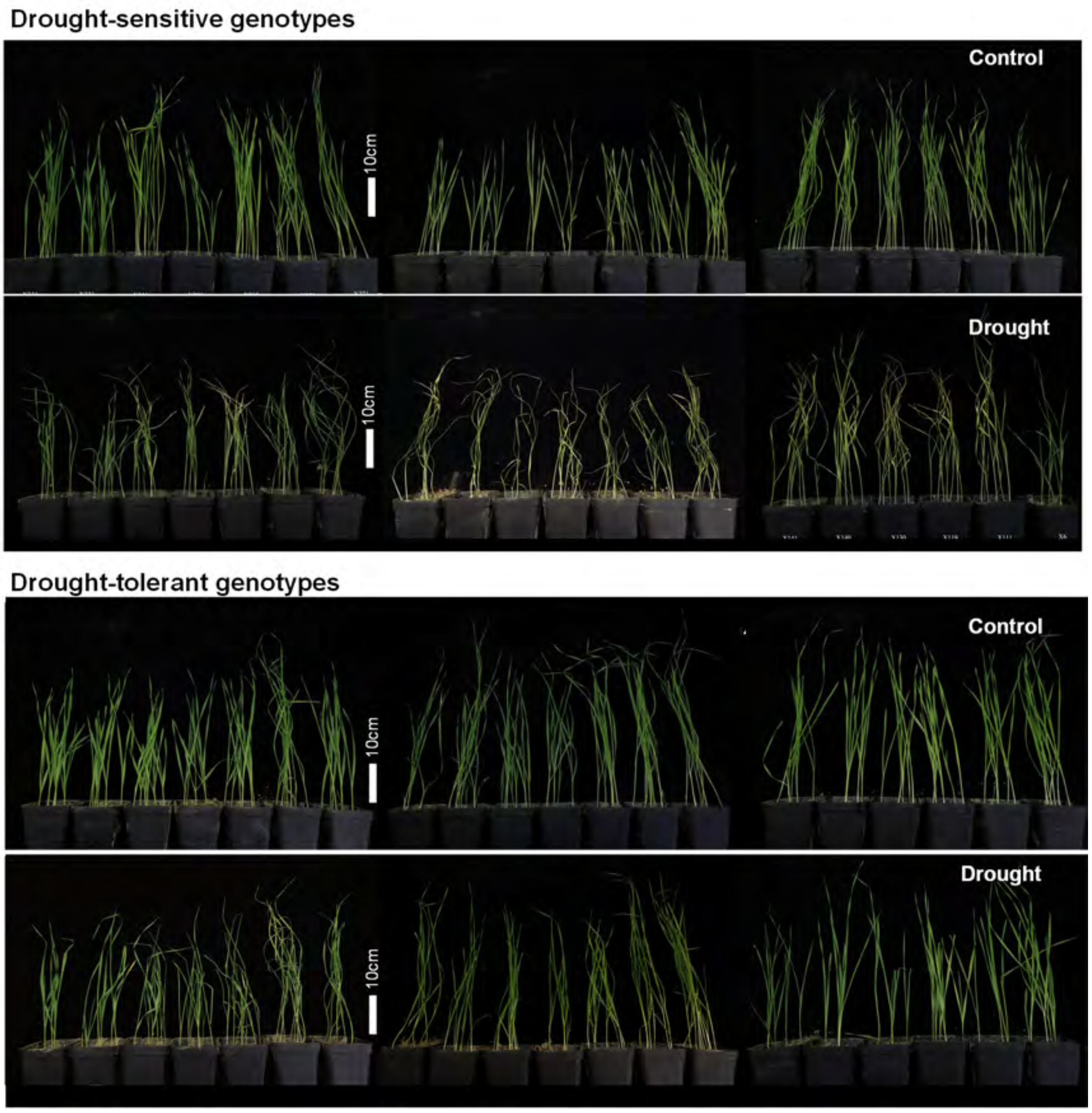

Figure S1. Seedling phenotypes of drought tolerant and sensitive wheat genotypes in the pot experiment one under drought treatment at SMC 4%.

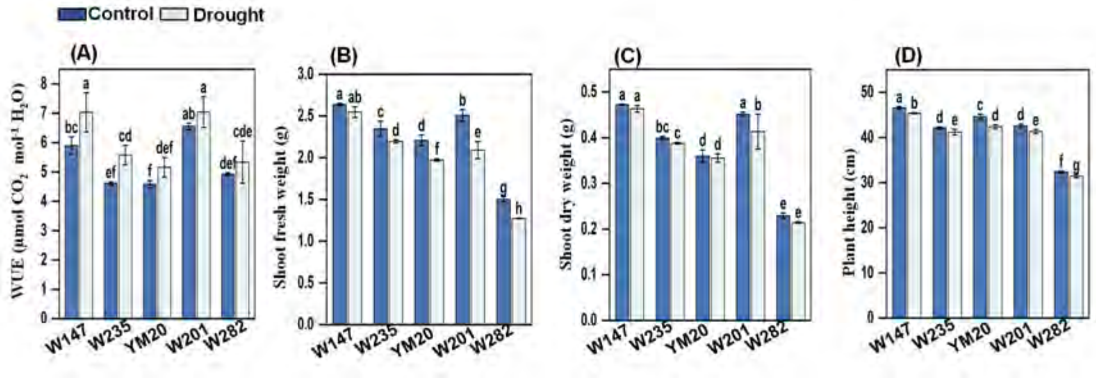

Figure S2. Effects of drought stress on agronomic traits and water metabolism of different wheat genotypes. (A) water use efficiencies (WUE), (B) shoot fresh weights, (C) shoot dry weight, (D) plant height. Data are means ± SEs (n = 3), different letters indicate significant differences at $P < 0.05$ according to Tukey's test.

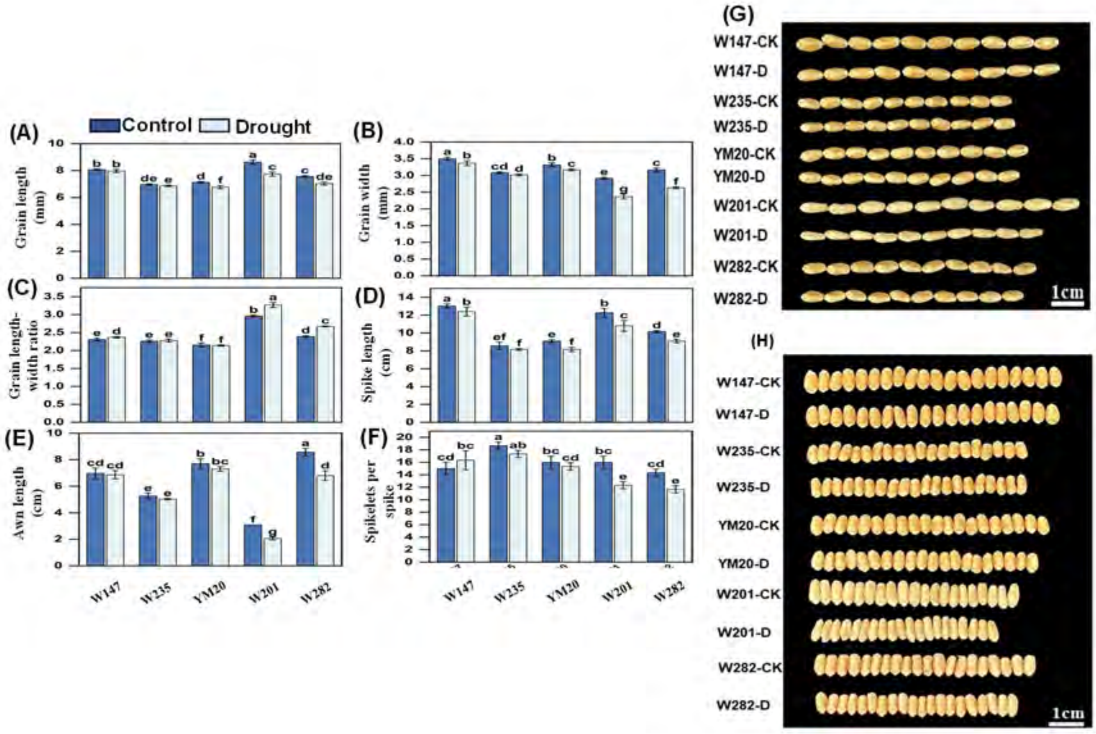

Figure S3. Effects of drought stress on yield parameters of different wheat genotypes. (A, G) Grain length; (B, H) Grain width; (C) Grain length-width ratio; (D) Spike length; (E) Awn length; (F) Spikelets per spike; Data are means ± SE (n=5), different letters indicate significant difference ($p < 0.05$).

Table S1 The detail information of 334 wheat core germplasms

Number	Name	Origin	Number	Name	Origin
W1	073/44	Australia	W168	H-142	China
W2	92FS-16	Australia	W169	H-143	China
W3	AEGYLOPS 14257	Australia	W170	H-144	China
W4	AFGHANISTAN 7	Australia	W171	H-145	China
W5	AHGAF	Australia	W172	H-146	China
W6	ALBIDUM 24	Australia	W173	H-147	China
W7	Al-wheat	Australia	W174	H-148	China
W8	ARTEMOVKA	Australia	W175	H-149	China
W9	AUS 19392	Australia	W176	H-150	China
W10	AUS 19393	Australia	W177	H-151	China
W11	AUS 19394	Australia	W178	H-152	China
W12	AUS 19395	Australia	W179	H-153	China
W13	AUS 19396	Australia	W180	H-154	China
W14	AUS 19397	Australia	W181	H-155	China
W15	AUS 19398	Australia	W182	H-157	China
W16	AUS 19399	Australia	W183	H-159	China
W17	AUS 19400	Australia	W184	H-160	China
W18	AUS 19401	Australia	W185	H-161	China
W19	AUS 19402	Australia	W186	H-162	China
W20	AUS 19403	Australia	W187	H-163	China
W21	AUS 19719	Australia	W188	H-164	China
W22	AUS 19720	Australia	W189	H-165	China
W23	B-T-17	Australia	W190	H-167	China
W24	B-T-35	Australia	W191	H-168	China
W25	B-T-38	Australia	W192	H-169	China
W26	B-T-51	Australia	W193	H-170	China
W27	BUKOVINKA	Australia	W194	H-171	China
W28	CAZ5 3	Australia	W195	H-172	China
W29	E.M.S. SUMMIT 70-8	Australia	W196	H-174	China
W30	EXITO B	Australia	W197	H-175	China
W31	FERRUGINEUM	Australia	W198	H-176	China
W32	FRETES	Australia	W199	H-177	China
W33	FRETES	Australia	W200	H-178	China
W34	FRETES	Australia	W201	H-179	China
W35	Glover	Australia	W202	H-180	China
W36	H-001	China	W203	H-181	China
W37	H-002	China	W204	H-183	China
W38	H-003	China	W205	H-184	China
W39	H-004	China	W206	H-185	China
W40	H-005	China	W207	H-186	China
W41	H-006	China	W208	H-187	China
W42	H-007	China	W209	H-189	China
W43	H-008	China	W210	H-190	China
W44	H-009	China	W211	H-191	China
W45	H-010	China	W212	H-192	China
W46	H-011	China	W213	H-193	China
W47	H-012	China	W214	H-194	China
W48	H-013	China	W215	H-195	China
W49	H-014	China	W216	H-196	China
W50	H-015	China	W217	H-197	China
W51	H-016	China	W218	H-198	China
W52	H-016	China	W219	H-199	China
W53	H-017	China	W220	H-200	China
W54	H-019	China	W221	H-201	China
W55	H-020	China	W222	H-202	China
W56	H-021	China	W223	H-203	China
W57	H-022	China	W224	H-204	China
W58	H-023	China	W225	H-205	China
W59	H-024	China	W226	H-206	China

Number	Name	Origin	Number	Name	Origin
W60	H-025	China	W227	H-207	China
W61	H-026	China	W228	H-208	China
W62	H-027	China	W229	H-209	China
W63	H-028	China	W230	H-210	China
W64	H-030	China	W231	H-211	China
W65	H-031	China	W232	H-212	China
W66	H-032	China	W233	H-213	China
W67	H-033	China	W234	H-214	China
W68	H-034	China	W235	H-215	China
W69	H-035	China	W236	H-216	China
W70	H-037	China	W237	H-217	China
W71	H-038	China	W238	H-218	China
W72	H-039	China	W239	H-219	China
W73	H-040	China	W240	H-220	China
W74	H-041	China	W241	H-221	China
W75	H-042	China	W242	H-222	China
W76	H-043	China	W243	H-223	China
W77	H-044	China	W244	H-224	China
W78	H-045	China	W245	H-225	China
W79	H-046	China	W246	H-226	China
W80	H-047	China	W247	H-227	China
W81	H-048	China	W248	H-228	China
W82	H-049	China	W249	H-229	China
W83	H-050	China	W250	H-230	China
W84	H-051	China	W251	H-231	China
W85	H-052	China	W252	H-232	China
W86	H-053	China	W253	H-233	China
W87	H-054	China	W254	H-234	China
W88	H-055	China	W255	H-235	China
W89	H-056	China	W256	H-236	China
W90	H-057	China	W257	H-237	China
W91	H-058	China	W258	H-238	China
W92	H-059	China	W259	H-239	China
W93	H-060	China	W260	H-240	China
W94	H-061	China	W261	H-241	China
W95	H-062	China	W262	H-242	China
W96	H-063	China	W263	H-243	China
W97	H-064	China	W264	H-244	China
W98	H-065	China	W265	H-245	China
W99	H-066	China	W266	H-246	China
W100	H-067	China	W267	H-247	China
W101	H-068	China	W268	H-249	China
W102	H-069	China	W269	H-250	China
W103	H-070	China	W270	H-251	China
W104	H-071	China	W271	H-252	China
W105	H-073	China	W272	H-253	China
W106	H-074	China	W273	Hartog	Australia
W107	H-075	China	W274	HB 88 I - 172	Australia
W108	H-076	China	W275	IG 43428	Australia
W109	H-077	China	W276	JAKUTIANKA 224	Australia
W110	H-078	China	W277	KARAGAN	Australia
W111	H-079	China	W278	KAZAKHSTANSKAJA 126	Australia
W112	H-080	China	W279	KHARCHIA 65	Australia
W113	H-081	China	W280	KOPARA 73	Australia
W114	H-082	China	W281	KORD CL PLUS	Australia
W115	H-083	China	W282	KZYL-SARK	Australia
W116	H-084	China	W283	LU 26S	Australia
W117	H-085	China	W284	MAHON DEMIAS	Australia
W118	H-086	China	W285	NAJAH	Australia
W119	H-087	China	W286	NAPOSTA	Australia
W120	H-088	China	W287	ONOHOISKAJA 4	Australia

Number	Name	Origin	Number	Name	Origin
W121	H-089	China	W288	PALESTINE 8	Australia
W122	H-090	China	W289	PI 177971	Australia
W123	H-091	China	W290	PI 178012	Australia
W124	H-092	China	W291	PI 178704	Australia
W125	H-093	China	W292	PI 180988	Australia
W126	H-094	China	W293	PI 264952	Australia
W127	H-095	China	W294	PI 94365	Australia
W128	H-096	China	W295	POBEDA	Australia
W129	H-097	China	W296	Preto Amarelo	Australia
W130	H-098	China	W297	Revenue	Australia
W131	H-099	China	W298	SEAGULL	Australia
W132	H-100	China	W299	SEL OF HOURANI 27	Australia
W133	H-101	China	W300	SEL OF HOURANI 27	Australia
W134	H-102	China	W301	SKALA	Australia
W135	H-103	China	W302	SOCIEDAD NACIONAL DE	Australia
W136	H-104	China	W303	SONNENWEIZEN	Australia
W137	H-105	China	W304	SURHAK 5688	Australia
W138	H-106	China	W305	SURHAK MESTNYJ	Australia
W139	H-107	China	W306	SW95 50101	Australia
W140	H-108	China	W307	SW95 50192	Australia
W141	H-109	China	W308	SW95 50213	Australia
W142	H-110	China	W309	SW95 50292	Australia
W143	H-111	China	W310	TURCICUM 1	Australia
W144	H-112	China	W311	VYSOKELITOVSKAYA	Australia
W145	H-113	China	W312	W 5013 EY-11-1	Australia
W146	H-114	China	W313	W 5228 HZ-1	Australia
W147	H-115	China	W314	W 5924 DT-34	Australia
W148	H-116	China	W315	wheat-2HBYDV	China
W149	H-117	China	W316	WL-wheat	China
W150	H-118	China	W317	Yang17	China
W151	H-119	China	W318	Yannong 15	China
W152	H-120	China	W319	Yannong 19	China
W153	H-121	China	W320	YRODY 1006	Australia
W154	H-122	China	W321	YU-01	China
W155	H-125	China	W322	YU-02	China
W156	H-126	China	W323	YU-03	China
W157	H-127	China	W324	YU-04	China
W158	H-129	China	W325	YU-05	China
W159	H-130	China	W326	YU-06	China
W160	H-131	China	W327	YU-07	China
W161	H-132	China	W328	YU-08	China
W162	H-133	China	W329	YU-09	China
W163	H-135	China	W330	YU-10	China
W164	H-136	China	W331	YU-11	China
W165	H-137	China	W332	YU-12	China
W166	H-138	China	W333	YU-13	China
W167	H-141	China	W334	ZHONG 4	China

Table S2 The twenty most drought-sensitive genotypes (ranked from most to least sensitive) and the twenty most tolerant genotypes (ranked from most to least tolerant) selected from the hydroponic-air drought simulation.

Drought-sensitive genotypes	Drought-tolerance genotypes
W201、W282、W130、W140、W271、W288、W272、W321、W119、W311、W111、W6、W331、W182、W172、W202、W141、W179、W261、W211	W147、W235、W267、W248、W328、W154、W315、W164、W239、W316、W126、W324、W236、W244、W223、W106、W287、W144、W313、W216

Table S3. Experiment Two. Effect of drought stress on growth and physiological traits of wheat plants in the pot experiment expressed as reduced (-)/increased (+) percentages of the well-watered controls.

**Drought tolerance index**	**W147**	**W235**	**W201**	**W282**	**YM20**	**$LSD_{0.05}$**
Thousand-kernel weight (g)	-5.41	-5.12	-15.33	-15.98	-8.42	3.90
Grains per spike	6.38	4.67	-11.71	0.53	-8.97	3.96
Spikes/plant	-27.14	-25.00	-33.33	-44.29	-50.00	1.00
Grain yield per plant (g)	-26.68	-26.58	-50.16	-52.94	-58.32	9.47
Tr	-27.31	-30.83	-37.32	-41.63	-35.94	7.28
Pn	-12.87	-18.73	-32.53	-36.75	-28.03	7.83
Ci	-2.04	-0.91	-3.93	-2.21	-2.67	1.58
Gs	-21.24	-26.81	-42.76	-29.52	-30.35	3.84
SPAD value	-5.47	-2.35	-16.43	-8.87	-10.26	1.66
Fv/Fm	-3.20	-1.03	-4.30	-1.39	-1.34	8.00
ΦPSII	-19.40	-10.00	-19.79	-25.63	-27.22	5.01
WSD	7.33	8.54	49.55	44.04	22.28	13.27
Free water	-1.40	-1.33	-2.82	-1.40	-2.32	0.96
Bound water	51.96	55.02	14.58	16.10	27.73	10.96
WUE	18.74	20.68	7.11	7.29	12.20	1.36
Plant height (cm)	-2.65	-2.14	-2.98	-2.98	-5.07	2.34
Shoot FW (g)	-3.45	-6.39	-16.59	-15.23	-10.64	3.43
Shoot DW(g)	-1.95	-2.87	-8.42	-6.47	-1.13	1.80
RWC (%)	-0.34	-0.76	-2.12	-1.86	-2.07	1.08
Integrated Score	-4.03	-3.81	-12.15	-11.62	-11.68	
